# Effects of the ant *Formica fusca* on the transmission of microsporidia infecting gypsy moth larvae

**DOI:** 10.1111/eea.12063

**Published:** 2013-04-20

**Authors:** Dörte Goertz, Gernot Hoch

**Affiliations:** 1 Department of Forest and Soil Sciences, BOKU – University of Natural Resources and Life SciencesHasenauerstraße 38, Vienna, 1190, Austria; 2 Department of Forest Protection, BFW – Federal Research Centre for ForestsSeckendorff-Gudent-Weg 8, Vienna, 1131, Austria

**Keywords:** *Lymantria dispar*, Hymenoptera, Formicidae, predation, *Nosema lymantriae*, *Vairimorpha disparis*, horizontal transmission, host-pathogen interaction, Burenellidae, Nosematidae, Lepidoptera, Erebidae

## Abstract

Transmission plays an integral part in the intimate relationship between a host insect and its pathogen that can be altered by abiotic or biotic factors. The latter include other pathogens, parasitoids, or predators. Ants are important species in food webs that act on various levels in a community structure. Their social behavior allows them to prey on and transport larger prey, or they can dismember the prey where it was found. Thereby they can also influence the horizontal transmission of a pathogen in its host's population. We tested the hypothesis that an ant species like *Formica fusca* L. (Hymenoptera: Formicidae) can affect the horizontal transmission of two microsporidian pathogens, *Nosema lymantriae* Weiser (Microsporidia: Nosematidae) and *Vairimorpha disparis* (Timofejeva) (Microsporidia: Burenellidae), infecting the gypsy moth, *Lymantria dispar* L. (Lepidoptera: Erebidae: Lymantriinae). Observational studies showed that uninfected and infected *L. dispar* larvae are potential prey items for *F. fusca*. Laboratory choice experiments led to the conclusion that *F. fusca* did not prefer *L. dispar* larvae infected with *N. lymantriae* and avoided *L. dispar* larvae infected with *V. disparis* over uninfected larvae when given the choice. Experiments carried out on small potted oak, *Quercus petraea* (Mattuschka) Liebl. (Fagaceae), saplings showed that predation of *F. fusca* on infected larvae did not significantly change the transmission of either microsporidian species to *L. dispar* test larvae. Microscopic examination indicated that *F. fusca* workers never became infected with *N. lymantriae* or *V. disparis* after feeding on infected prey.

## Introduction

The transmission of a pathogen is a vitally important aspect of the interaction of a host and its pathogen. Pathogens like microsporidia are transmitted horizontally, vertically, or both, depending on the species (Becnel & Andreadis, 1999). Horizontal transmission is the transfer of pathogens from one host to another (Steinhaus & Martignoni, 1970), vertical transmission is the transfer of the pathogen from one generation to the next (Becnel & Andreadis, 1999). Horizontal transmission can be achieved either directly from an infectious to a susceptible host by the release of free-living pathogenic stages from the infectious host into the environment, or indirectly by vectors such as parasitic wasps or by predators that feed on infectious prey. When a predatory insect encounters a prey insect infected with an entomopathogen, the pathogen can have direct (via infection) or indirect effects on the predator (van Essen & Anthony, 1976; Sajap et al., 1999; Lee & Fuxa, 2000). For example, a predator may make prey choices based on whether or not the prey is infected (Bell et al., 2004). When infected prey is not avoided, the predator may contribute to dissemination of inoculum in the environment or to removal of inoculum (Down et al., 2004). Both possibilities have important consequences for transmission of a pathogen in the population of the natural host. A previous study showed that activity of the specialized predatory beetle *Calosoma sycophanta* L. (Coleoptera: Carabidae) can lead to increased transmission of a microsporidian pathogen in its prey, the gypsy moth, *Lymantria dispar* L. (Lepidoptera: Erebidae: Lymantriinae) (Goertz & Hoch, 2013).

The gypsy moth is a well-known forest defoliator throughout the northern hemisphere. Eggs hatch in April and larvae begin feeding on leaves of deciduous trees, in Europe preferentially on oak species. They molt 4–5 times until pupation in late spring and early summer. In Central Europe, all larval stages are hosts and prey for a number of pathogens, parasites, parasitoids, or predators, for example, the predatory beetle *C. sycophanta*, the parasitic wasp *Glyptapanteles liparidis* (Bouché), or pathogens like a nucleopolyhedrosis virus and several microsporidian species (Hoch et al., 2001).

*Nosema lymantriae* Weiser (Microsporidia: Nosematidae) and *Vairimorpha disparis* (Timofejeva) (Microsporidia: Burenellidae) are two microsporidian species that infect *L. dispar* larvae. Following the ingestion of environmental spores by a susceptible larva, both pathogens undergo a first developmental cycle in the midgut of the host larva resulting in the production of primary spores. These spores germinate inside the host and thereby spread the infection to cells of the target tissues, in case of *N. lymantriae* silk glands, fat body, gonads, and Malpighian tubules, in case of *V. disparis* the fat body. The first environmentally stable spores of *N. lymantriae* and *V. disparis* that are infectious to other *L. dispar* larvae are recorded, respectively, in the silk glands 9 days post infection (dpi) and in the fat body 7 dpi. Although these target tissues are filled with spores by 10–12 dpi, the larvae are not yet infectious because they do not release spores into the environment at this point. Following this latent period of the larva of about 2 weeks, horizontal transmission of *N. lymantriae* starts when the Malpighian tubules are filled with environmental spores that are released continuously with feces by the infectious larva. An infection with *N. lymantriae* finally leads to the death of the larva after about 4 weeks of infection; spores are released from the decomposing cadaver. In contrast, horizontal transmission of *V. disparis* takes only place after host death, about 4 weeks after infection when environmental spores are released from the decomposing cadaver (Goertz & Hoch, 2008).

Ants are important species in various food webs and ecosystems. They act on various levels in a community structure either by being primary or secondary predators, that is preying on herbivores or other predatory arthropods, and by being facilitators of mutualistic herbivores (Mooney & Tillberg, 2005). Their social behavior allows them to prey on and transport larger prey that is not acceptable for other predators like wasps or bugs (Dyer, 1997; Robson & Traniello, 1997). Several studies documented the importance of wood ants, *Formica* spp., as predators of lepidopteran larvae (Horstmann, 1970; Weseloh, 1989; Puntilla et al., 2004; Mooney & Tillberg, 2005; Aimi et al., 2008). Red wood ants (*Formica polyctena* Forster group) cleaned *Pinus* trees surrounding their nest of newly hatched and young instars of the noctuid moth *Panolis flammea* Denis & Schiffermüller within 10 days during an outbreak (Behrndt, 1933). Forest ants are also known to prey on *L. dispar* early instars or pupae (Smith & Lautenschlager, 1981; Weseloh, 1989). Ants of the genus *Formica* seem to be the most important ant predators of young gypsy moth larvae in the northeastern USA due to their high abundance and predatory activity (Weseloh, 1989). Our own observations during a recent field experiment suggested that later instars of *L. dispar* infected with microsporidia could be foraged and dismembered by ants possibly impacting microsporidia transmission (D Goertz & G Hoch, unpubl.).

*Formica fusca* L. (Hymenoptera: Formicidae), the common black ant, is a paleartic, polygynous, and thermophilic species that builds nests with no more than 500–2 000 workers and up to 15 queens into the soil below stones, broken trees, or branches in open forests. The workers feed on insects, honey dew, and extrafloral nectaries (Seifert, 2007). We used *F. fusca* as model species for predatory ants because it occurs in the natural *L. dispar* habitat, preys on *L. dispar* larvae, and is easy to rear in the laboratory.

The aim of this study was to test whether an ant species like *F. fusca* can influence the horizontal transmission of microsporidian pathogens infecting *L. dispar*. In a first observational study we tested whether various ant species living in the natural habitat of *L. dispar* prey on infected or uninfected larvae or cadavers. Furthermore, we tested whether *F. fusca* discriminates between uninfected and infected prey and whether it disseminates spores during predation. Moreover, we used experiments with caged and potted oak saplings to test whether *F. fusca* can influence the horizontal transmission of *N. lymantriae* or *V. disparis*.

## Materials and methods

### Insects and pathogens

*Lymantria dispar* larvae of the New Jersey Standard Strain were used as hosts for two microsporidian pathogens, *N. lymantriae* and *V. disparis*, and prey for *F. fusca*. Egg masses were obtained from the USDA-APHIS Otis Method Development Center (Otis, MA, USA). *Lymantria dispar* larvae reared from these egg masses were regularly examined microscopically and confirmed to be free from microsporidian infection. If not indicated otherwise, larvae were reared at L16(24 °C):8D(18 °C) photo- and thermoperiod. They were kept individually or in small groups in plastic cups with a volume of 50 or 250 ml, respectively, on a wheat germ diet (Bell et al., 1981).

*Formica fusca* colonies were collected from mixed hardwood forests, with *Quercus petraea* (Mattuschka) Liebl. (Fagaceae) as dominant species, where *L. dispar* is known to occur. Nests with about 100 workers and one queen were created and placed into one nest box. Large colonies with more than one queen and more than 100 workers per queen were divided and placed into two or more nest boxes. The nests were placed into plastic boxes (20 ×20 × 14 cm) with ventilated lids and plastered floor and provided with tissue paper as nest material. The ants were reared at 21 °C and L16:D8 photoperiod and hibernated at 12 °C and L8:D16h. Honey-water solution, insects (first and second instar *L. dispar*), and water were provided as food.

The two microsporidian species, *N. lymantriae* (accession no. 1996-A) and *V. disparis* (accession no. 1995-D), were used for all experiments. The *N. lymantriae* isolate originated from silk glands of *L. dispar* larvae, collected in 1996 near Levishte, Bulgaria. *Vairimorpha disparis* was originally isolated from fat bodies of *L. dispar* larvae, collected in 1995 near Rupite, Bulgaria. Both microsporidian species are stored with the accession number mentioned above, and were obtained from, the germ-plasma collection of the Illinois Natural History Survey (Urbana-Champaign, IL, USA; laboratory of Dr LF Solter). Microsporidian spores were produced in *L. dispar* larvae as described in Goertz & Hoch (2008). The spore suspensions were stored as 1:1 mixtures with glycerol in liquid nitrogen up to 3 months until use in the experiments.

For all experiments, controlled infections with the different microsporidian parasites were produced following the protocol used in our laboratory (Goertz & Hoch, 2008). Newly molted third instars were starved for 24 h followed by individual inoculation of the larvae in 24-well tissue culture plates with 1-μl spore suspension containing 10^3^ spores μl^−1^ pipetted onto a 2-mm^3^ diet block. Only larvae that ingested the entire diet block within 24 h were used for the experiments. Control larvae were treated in the same manner, but the diet blocks were inoculated with distilled water.

At the end of each experiment, all *L. dispar* individuals (larvae, pupae, or adults) were microscopically examined for the presence of microsporidia. Cross sections of each insect were prepared and inspected under phase contrast microscopy at 200–400× magnification. Additionally, all *F. fusca* workers, alates, and queens were dissected and inspected for an infection with *N. lymantriae* or *V. disparis* after the end of all experiments.

### Observational field study

Field experiments were carried out in mixed oak forests in which *L. dispar* naturally occurs to determine whether ants that forage in this habitat, could influence the transmission of microsporidia. Therefore, five study sites were selected in forests at the western outskirts of Vienna, Austria (48°15′N, 16°17′E). Two uninfected larvae, two live larvae infected with *N. lymantriae*, and two cadavers infected with *N. lymantriae* were simultaneously placed in an open Petri dish near ant trails on the ground of an oak forest. Offered larvae and any approaching ants were observed for 15 min and then removed. Afterwards, two uninfected larvae, two live larvae infected with *V. disparis*, and two cadavers infected with *V. disparis* were offered in a new open Petri dish near ant trails on the ground of the oak forest and observed for 15 min. All larvae and cadavers were in the fourth or fifth instar. During the observational period, the following parameters were recorded: time between exposure of *L. dispar* and first detection of *L. dispar* by ant workers, total number of workers at the exposed prey, number of *L. dispar* removed by the ants, and reaction of workers to the offered larvae. The reaction of workers was assessed according to four categories: (1) ignored – no reaction to the offered prey; (2) visited – larvae were antennated; (3) attacked – workers tried to overwhelm the live larvae or to remove the cadavers from the Petri dish; and (4) foraged – successful removal of larvae or cadavers from the Petri dish. A sample of ant workers was collected for further identification of the ant species using the key by Seifert (2007). The observational studies were carried out in June between 09:00 and 16:00 hours under sunny weather conditions when air temperatures were about 25 °C. A Kruskal–Wallis H-test was applied to examine differences in arrival time and number of workers at the Petri dish between ant species as data did not follow a normal distribution. All statistical analyses were done with SPSS version 18 (IBM, Somers, NY, USA).

### Discrimination between infected and uninfected prey by *Formica fusca*

Colonies of *F. fusca* containing about 100 workers and one queen were allowed to choose between uninfected and infected *L. dispar* larvae. Two uninfected and two infected *L. dispar* larvae of the same size and age (at 16 or 17 dpi, when inoculated larvae became infectious and had molted into the fourth or fifth instar) were placed into the arenas of nest boxes. After 24 h, all remaining larvae and larval cadavers were counted, removed, and checked microscopically for the status of infection. The experiment was repeated 22 and 23 times with new groups of ants for *Vairimorpha-*infected or *Nosema-*infected larvae, respectively. Log ratios (LR) as described by Roy et al. (2008) were calculated to test for the preference of uninfected or infected *L. dispar* larvae: LR = log [(i + 0.05)/(u + 0.05)], where i and u are the numbers of foraged infected and uninfected larvae, respectively. A value of zero indicates no preference, a negative value shows a preference for uninfected, and a positive one a preference for infected larvae. A one-sample t-test was used to test for statistically significant differences from zero.

### Dissemination of spores by *Formica fusca* when preying on *Lymantria dispar*

Newly molted *L. dispar* third instars were inoculated with either *N. lymantriae* or *V. disparis* as described above. At 16 or 17 dpi, five larvae inoculated with the same microsporidian species were placed onto oak foliage bouquets in each of 12 rearing cages (29 cm high, 20 cm diameter). They were allowed to acclimate for 2 h. Then, six cages (three with *N. lymantriae* infected larvae, three with *V. disparis* infected larvae) were individually connected to laboratory colony nest boxes of *F. fusca* by a flexible, clear plastic tube (60 cm long, 15 mm diameter). Workers of *F. fusca* were allowed to prey on the inoculated larvae for 24 h. *Lymantria dispar* larvae of the remaining six cages were undisturbed. After 24 h, all ants, inoculated larvae, and larval cadavers were removed from the cages and 10 susceptible, newly molted *L. dispar* third instars were placed into the cages and allowed to feed on the potentially contaminated oak leaves for a period of 3 days. Following the exposure period, all test larvae were reared individually on wheat germ diet for 20 days to allow any acquired infection to develop. All test larvae were dissected at the end of the experiment and checked for the presence of microsporidian spores. The percent infection was recorded. The experiment was repeated six times giving a total of 18 replicates. A Mann–Whitney U-test was applied to examine differences in infection between treatments as data did not follow a normal distribution according to Kolmogorov–Smirnoff tests.

### Effects of *Formica fusca* on the horizontal transmission of microsporidia

**Figure 1:**
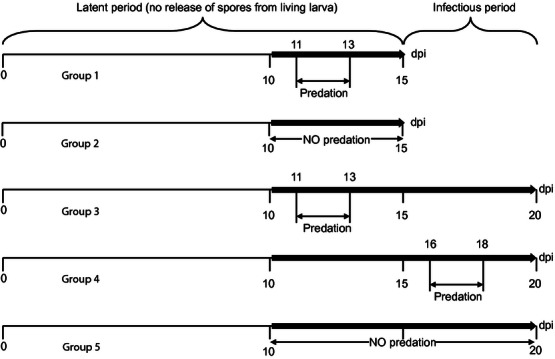
Schematic of the experimental design performed to test the influence of *Formica fusca* on the horizontal transmission of either *Nosema lymantriae* or *Vairimorpha disparis*. The exposure periods (thick arrow) which always began at 10 dpi and the predation periods of *F. fusca* for each treatment group are indicated. Each group was set up in eight replicates. For further details refer to the text.

To test whether workers of *F. fusca* can influence the transmission of *N. lymantriae* or *V. disparis* under more natural conditions the following experiment was conducted in 2008 and 2009 with a total of eight replicates for each treatment and microsporidian species. Newly molted *L. dispar* third instars were individually inoculated with either *N. lymantriae* or *V. disparis* as described above. They were reared on wheat germ diet until 5 dpi and then on foliage of *Q. petraea*. All inoculated larvae were permanently marked by clipping one proleg 2 dpi. This procedure does not increase mortality among larvae and does not reduce their mobility (Weseloh, 1985; Hoch et al., 2008). Ten days post infection, nine infected and marked *L. dispar* larvae and 21 uninfected newly molted third instars (= test larvae) were placed onto potted 2-year-old and 1-m-high *Q. petraea* saplings. This prevalence of 30% infected insects was chosen based on previous studies examining horizontal transmission (Goertz & Hoch, 2009). The plants were divided into five groups (Figure 1). Larvae of the first two groups were allowed to feed together for 5 days (10–15 dpi). At 11 dpi, that is during the latent period of the microsporidian infection, nest boxes with laboratory colonies of *F. fusca* were connected by a flexible plastic tube (60 cm long, 15 mm diameter) with each cage of the first group and workers of the colonies were allowed to prey inoculated and test larvae for 48 h. The larvae of the second group were undisturbed. *Lymantria dispar* larvae of the remaining three groups were reared together for 10 days (10–20 dpi). Laboratory colonies of *F. fusca* were connected by a flexible plastic tube with each cage of the third group for 48 h at 11 dpi, and with each cage of the fourth group at 16 dpi (just after the beginning of the infectious period); larvae of the fifth group were undisturbed. In addition, 30 uninfected newly molted third instars were placed onto additional oak plants and held for 5 and 10 days as negative controls. All potted oak plants were placed into mesh bags (1 mm^2^ mesh). The bags were fixed to the pots with double-sided adhesive tape preventing predation or parasitization by other insects. All test larvae were reared individually after the exposure period on diet for 20 days to allow any acquired infection to develop. All larvae were dissected at the end of the experiment to determine their status of infection.

The datasets of multiple dependent scale variables of this experiment were analyzed by MANOVA using the GLM multivariate procedure of SPSS, testing the effects of the factors ‘exposure period of larvae’ and ‘predation period of ants’ on the dependent variables ‘percent infection’ (i.e.,% test larvae that became infected), ‘recovery rate of inoculated larvae’, and ‘recovery rate of test larvae’. Frequency datasets were arcsin(√p) transformed and Box's M was used to test the null hypothesis that the observed covariance matrices of the dependent variables were equal across groups.

## Results

### Observational field study

In total, 10 ant species were tested during 84 observation periods. The number of observation periods varied between four and 36 per site. *Dolichoderus quadripunctatus* (L.) arrived at the Petri dish containing exposed larvae between 1–3 min after the beginning of the observation period (Table 1). Workers attacked all live larvae and ignored cadavers and up to six workers were observed attacking. *Formica cunicularia* Latreille attacked only infected larvae and between 25 and 33% of the attacks were successful. Control larvae and cadavers of infected larvae were not attacked. First workers of *F. fusca* arrived after 4.3 ± 2.9 min and on average 1.6 workers were recorded per larva. Up to nine workers preyed upon a single infected or uninfected *L. dispar* larva at one time. All prey types (cadaver, infected larvae, uninfected larvae) were attacked. *Formica gagates* Latreille was also observed at the Petri dishes, but never attacked any *L. dispar*. Workers of *F. polyctena* arrived after 3.4 ± 2.6 min at the Petri dish and attacked all prey types offered, but removed only cadavers. Up to 17 *F. polyctena* were observed preying upon a single *L. dispar* larva infected with *N. lymantriae*. *Lasius emarginatus* (Olivier) arrived and preyed upon all offered prey types after about 4 min. Initially workers of *Lasius fuliginosus* (Latreille) approached uninfected and infected *L. dispar* larvae or cadavers about 1 min earlier and attacked only live *L. dispar*. Up to 16 workers of *L. emarginatus* or *L. fuliginosus* were observed attacking a single larva infected with *V. disparis*. No overall significant differences were found in the mean number of workers of the different ant species at the exposed larvae (H-test: χ^2^ = 20.25, d.f. = 8, P = 0.09) or their time of arrival (χ^2^ = 11.58, d.f. = 8, P = 0.17).

**Table 1:** Observations on foraging ant species, offered a Petri dish with uninfected *Lymantria dispar* larvae (control), live larvae infected either with *Nosema lymantriae* or *Vairimorpha disparis*, and larval cadavers containing spores of either microsporidian species. Mean (± SD) time to arrival of ants at the prey, mean number (+ range) of ant workers arriving, number of attacks (+ successful attacks in parentheses), and number of observations (i.e., Petri dish replicates) are given

Ant species	Arrival at prey (min)	No. workers at prey		No. attacks (successes)	No. observations

		Mean	Range		
*Dolichoderus quadripunctatus*	2.0 ± 1.4	1.2	0–6	3 (0)	4
Control	2.3 ± 1.8	1.0	0–3	1 (0)	
*N. lymantriae*	3.5	1.0	0–3	1 (0)	
*N. lymantriae* (cadaver)	–	0		–	
*V. disparis*	1.0 ± 0.0	2.3	0–6	1 (0)	
*V. disparis* (cadaver)	–	0		–	
*Formica cunicularia*	2.4 ± 2.1	2.8	0–12	7 (2)	12
Control	1.0 ± 0.0	1.5	0–3	0 (0)	
*N. lymantriae*	2.8 ± 2.5	2.5	0–7	4 (1)	
*N. lymantriae* (cadaver)	–	0		–	
*V. disparis*	2.4 ± 2.1	4.3	0–12	3 (1)	
*V. disparis* (cadaver)	–	0		–	
*Formica fusca*	4.3 ± 2.9	1.6	0–9	9 (2)	14
Control	5.7 ± 3.9	0.8	0–4	1 (0)	
*N. lymantriae*	3.4 ± 2.6	1.8	0–9	2 (0)	
*N. lymantriae* (cadaver)	3.6 ± 2.9	2.1	0–8	2 (1)	
*V. disparis*	5.6 ± 2.8	1.2	0–7	2 (0)	
*V. disparis* (cadaver)	3.6 ± 2.9	2.1	0–8	2 (1)	
*Formica gagates*	4.0 ± 4.8	4.4	2–7	0 (0)	4
*N. lymantriae*	5.7 ± 7.7	4.0	2–6	0 (0)	
*N. lymantriae* (cadaver)	0.3	2.0	2–2	0 (0)	
*V. disparis*	2.4 ± 0.4	6.0	5–7	0 (0)	
*Formica polyctena*	3.4 ± 2.6	3.3	0–17	11 (3)	18
Control	4.1 ± 2.4	2.0	0–4	3 (0)	
*N. lymantriae*	2.6 ± 1.5	4.8	1–17	4 (0)	
*N. lymantriae* (cadaver)	2.0	2.0	0–4	1 (1)	
*V. disparis*	2.0 ± 1.5	2.7	0–7	1 (0)	
*V. disparis* (cadaver)	6.5 ± 6.4	6.5	1–12	2 (2)	
*Lasius emarginatus*	4.1 ± 2.4	3.6	0–16	7 (2)	10
Control	2.5 ± 2.1	1.3	0–6	1 (0)	
*N. lymantriae*	5.3 ± 3.2	1.0	0–2	1 (0)	
*N. lymantriae* (cadaver)	4.0 ± 1.0	8.7	2–14	2 (0)	
*V. disparis*	5.0 ± 3.6	3.8	0–16	1 (1)	
*V. disparis* (cadaver)	3.3 ± 2.1	7.0	2–10	2 (1)	
*Lasius fuliginosus*	3.2 ± 3.8	4.2	0–16	10 (1)	14
Control	3.4 ± 4.2	4.8	2–13	2 (0)	
*N. lymantriae*	3.8 ± 4.1	4.3	2–7	4 (1)	
*N. lymantriae* (cadaver)	–	0		–	
*V. disparis*	2.2 ± 3.8	6.3	0–16	4 (0)	
*V. disparis* (cadaver)	–	0		–	
*Lasius alienus*	1.0	2.0	0–4	0 (0)	1
*V. disparis*	1.0	4.0	4–4	0 (0)	
*V. disparis* (cadaver)	–	0		–	
*Lasius niger*	1.4 ± 0.6	3.4	0–11	5 (4)	6
Control	–	0		–	
*N. lymantriae*	1.0	3.7	0–11	1 (1)	
*N. lymantriae* (cadaver)	2.0	3.0	0–9	1 (1)	
*V. disparis*	1.0	3.7	0–11	1 (0)	
*V. disparis* (cadaver)	1.5 ± 0.7	6.7	0–11	2 (2)	
*Leptothorax gredleri*	–	0		–	1
Control	–	0		–	
*N. lymantriae*	–	0		–	
*N. lymantriae* (cadaver)	–	0		–	
*V. disparis*	–	0		–	
*V. disparis* (cadaver)	–	0		–	

### Discrimination between infected and uninfected prey by *Formica fusca*

When *F. fusca* were offered two uninfected *L. dispar* larvae and two larvae infected with *V. disparis*, workers of *F. fusca* transported on average 0.27 ± 0.55 infected and 0.73 ± 0.88 uninfected larvae into the nest within the 24 h of exposure. Calculated log ratios indicated that uninfected larvae were preferred over *Vairimorpha*-infected ones (Table 2). Workers of *F. fusca* transported on average more uninfected than *Nosema*-infected larvae into their nests; however, this preference was not significant (Table 2). No *F. fusca* worker or queen was infected with *N. lymantriae* or *V. disparis*.

**Table 2:** Mean (± SD) number of *Lymantria dispar* larvae foraged by *Formica fusca* workers, log ratio describing preference, and results of one-sample t-test testing deviation of log ratio from zero

Prey	No. larvae	Log ratio	t	d.f.	P
*Nosema lymantriae*					
Infected	0.57 ± 0.79	−0.22	−1.1611	22	0.36
Uninfected	0.74 ± 0.75				
*Vairimorpha disparis*					
Infected	0.27 ± 0.55	−0.37	−2.2653	21	0.03
Uninfected	0.73 ± 0.88				

### Dissemination of spores while preying on infected larvae

**Figure 2:**
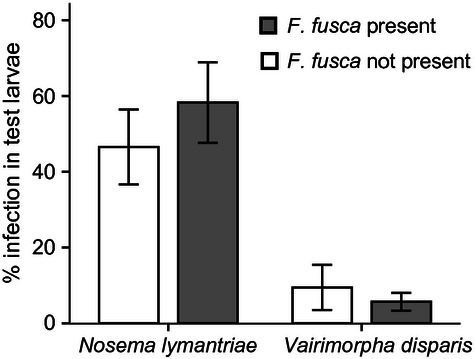
Mean (± SE) percent infection of *Lymantria dispar* test larvae feeding on foliage that was possibly contaminated with spores of *Nosema lymantriae* or *Vairimorpha disparis* following predation by *Formica fusca* on microsporidia-infected *L. dispar* larvae. Infection rate did not differ significantly between treatments with and without ants (U-test: P>0.05).

When *F. fusca* was allowed to prey on *L. dispar* larvae either infected with *V. disparis* or *N. lymantriae*, the presence of *F. fusca* had no effect on the transmission of either microsporidian species to test larvae exposed afterwards (Figure 2). In case of *V. disparis*, 5 and 8% new infections were recorded in *L. dispar* test larvae, with *F. fusca* present or not, respectively; this difference was not significant (U-test: U = 234.5, P = 0.62). The proportion of new infections with *N. lymantriae* in test larvae increased insignificantly by 10%, when workers of *F. fusca* had been allowed to prey on inoculated *L. dispar* larvae earlier (U = 258.5, P = 0.33; Figure 2). *Formica fusca* workers or queens were not infected with *N. lymantriae* or *V. disparis*.

### Effects of *Formica fusca* on the horizontal transmission of microsporidia

#### Nosema lymantriae

**Figure 3:**
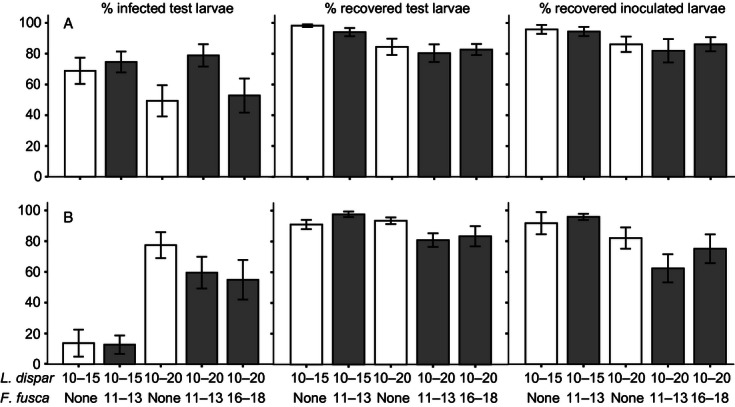
Influence of *Formica fusca* on the mean (± SE) percent infection of *Lymantria dispar* test larvae with (A) *Nosema lymantriae* or (B) *Vairimorpha disparis*, their recovery rates, and the proportions of recovered inoculated larvae. White bars represent the ‘no-ant’ control with short (10–15 dpi) and long (10–20 dpi) larval exposure period; gray bars represent treatments with short (10–15 dpi) and long (10–20 dpi) exposure period of *L. dispar* and with *F. fusca* workers present either 11–13 or 16–18 dpi. *Formica fusca* did not influence any dependent variable. For detailed statistical analysis see Table 3.

Between 80.4 and 98.2% of the test and inoculated larvae were recovered at the end of the exposure periods. Of the test larvae, between 49.4 and 78.9% became infected with *N. lymantriae*. No effect of ant predation or experimental period was measured on the transmission of *N. lymantriae*. The presence of *F. fusca* did not influence percent infection of test larvae (P = 0.14), recovery of test larvae (P = 0.43), or inoculated larvae (P = 0.75) (MANOVA; Table 3, Figure 3). The experimental period did affect recovery of test larvae (P<0.001) and of inoculated larvae (P = 0.04), leading to lower recovery after longer exposure. No significant interaction effect of experimental period and ant predation existed for any of the three tested dependent variables.

**Table 3:** Results of MANOVA for the horizontal transmission experiments with *Nosema lymantriae* or *Vairimorpha disparis* testing the effects of the factors (and their interaction) ‘exposure period’ of *Lymantria dispar* larvae in the cages (two levels: 10–15 and 10–20 dpi) and ‘*F. fusca*’ (three levels: no *Formica fusca* present, ants present early, and ants present late), on the dependent variables ‘% infection of test larvae’, ‘% recovered test larvae’, and ‘% recovered inoculated larvae’

Factor	Dependent variable	MS	d.f.	F	P	Partial η^2^
*Nosema lymantriae*						
Exposure period	% infected test larvae	0.056	1	0.57	0.45	0.016
	% recovered test larvae	0.697	1	23.36	<0.001	0.400
	% recovered inoculated larvae	0.298	1	4.48	0.04	0.113
*F. fusca*	% infected test larvae	0.224	1	2.28	0.14	0.061
	% recovered test larvae	0.019	1	0.65	0.43	0.018
	% recovered inoculated larvae	0.007	1	0.10	0.75	0.003
Exposure period × *F*. *fusca*	% infected test larvae	0.045	1	0.44	0.51	0.012
	% recovered test larvae	0.000	1	0.00	0.99	0.000
	% recovered inoculated larvae	0.001	1	0.01	0.91	0.000
*Vairimorpha disparis*						
Exposure period	% infected test larvae	4.668	1	42.697	<0.001	0.564
	% recovered test larvae	0.091	1	2.043	0.16	0.058
	% recovered inoculated larvae	0.698	1	6.555	0.02	0.166
*F. fusca*	% infected test larvae	0.115	1	1.048	0.31	0.031
	% recovered test larvae	0.002	1	0.051	0.82	0.002
	% recovered inoculated larvae	0.021	1	0.201	0.66	0.006
Exposure period × *F*. *fusca*	% infected test larvae	0.453	1	4.573	0.40	0.022
	% recovered test larvae	0.296	1	8.255	0.07	0.020
	% recovered inoculated larvae	0.236	1	2.367	0.13	0.067

*Nosema lymantriae*: Box's M = 26.67, d.f. = 24, P = 0.62; *Vairimorpha disparis*: M = 34.14, d.f. = 24, P = 0.32. All data transformed.

#### Vairimorpha disparis

At least 80.9% of the test larvae and between 62.4 and 95.8% of the inoculated larvae were recovered at the end of the exposure periods. When *F. fusca* was allowed to prey upon *L. dispar* larvae during the long exposure period (10–20 dpi), the proportion of infected test larvae decreased by more than 17%. Nevertheless, the presence of *F. fusca* did not significantly influence the percent infection with *V. disparis* in test larvae (P = 0.31) or recovery of test (P = 0.82) or inoculated (P = 0.66) larvae. A longer experimental period caused a higher proportion of test larvae to become infected (P<0.001) and lower recovery of inoculated larvae (P = 0.015) (MANOVA; Table 3, Figure 3); the proportion of infected test larvae increased by 41%. No interaction between presence of *F. fusca* and experimental period was found. None of the workers or queens of *F. fusca* was infected with either microsporidian species at the end of the experiment.

## Discussion

*Formica fusca* is common in the natural habitat of *L. dispar* (Seifert, 2007). The observational study showed that in many cases (25%) *F. fusca* was the ant species that preyed on the offered *L. dispar* larvae. Workers attacked all prey types, that is infected and uninfected larvae as well as spore-containing cadavers. In addition, *F. fusca* workers removed cadavers during the short observational period of 15 min. This shows that the model organism we chose for our laboratory experiments does occur in the habitat of *L. dispar* and does prey on *L. dispar* larvae. Other species, such as *Lasius fuliginosus*, *L. niger*, or *F. cunicularia* avoided control larvae, cadavers, and larvae infected with one of the tested microsporidia. The behavioral reactions of the observed ant species to the presented prey indicate that the potential effects of a given ant species on the transmission of microsporidia vary and no generalizations should be made.

Workers, queens, and alates of *F. fusca* never became infected by either microsporidian species and no other negative effects on the colony were observed. There are several factors that might explain the failure of either microsporidian species to infect workers or alates of *F. fusca*. The ejection of the polar filament, therefore the possible successful infection of the host's midgut cells, depends strongly on several stimuli as pH or ion concentrations that might be different between workers of *F. fusca* and *L. dispar* larvae resulting in resistance of *F. fusca* to both microsporidian species (Undeen, 1990; Chapman, 1998; Cali & Takvorian, 1999). Furthermore, it is not clear whether spores of *N. lymantriae* or *V. disparis* pass the buccal tube and the infrabuccal chamber. Both structures form a very efficient filtering system in the fire ant *Solenopsis invicta* Buren, filtering out all particles greater than 0.75 μm and therefore preventing ingestion of *Thelohania solenopsae* Knell, Allen & Hazard spores (Oi, 2006). Spores of *N. lymantriae* (2–2.5 μm wide; Weiser, 1957, 1998) and *V. disparis* (2.6 μm; Vavra et al., 2006) would likewise be unable to pass such a filter. Following a separation of liquids contained in the ingested food particles of varying size, the solid residuum formed in the infrabuccal chamber is eventually thrown out by the worker ant (Wheeler, 1925). Therefore, we conclude that adult *F. fusca* are not negatively affected when they prey on *Nosema*- or *Vairimorpha*-infected larvae.

The social behavior of ants gives workers several options when preying on other arthropods: they can either transport the intact food item to the nest alone, recruit other workers and together transport the prey to the nest, or they can dismember the prey where it was found (Dyer, 1997; Robson & Traniello, 1997). The first two options would probably lead to the removal of inoculum from the host's environment and therefore lead to a lower transmission of the pathogens. The last option might increase the transmission of the pathogens due to dispersal of spores into the host's environment. Our results do not indicate that transmission of *N. lymantriae* is either enhanced or decreased by the action of *F. fusca*. Infected larvae were not avoided or preferred over uninfected ones. When *F. fusca* was allowed to prey on infected larvae in small cages, the percent infection of test larvae was not significantly different from the undisturbed treatment and no higher or lower transmission of *N. lymantriae* was observed under more natural conditions when *F. fusca* was allowed to prey either on infectious *L. dispar* larvae or uninfected test larvae. Similarly, *C. sycophanta*, a predatory beetle of *L. dispar* larvae that is able to disseminate spores of *N. lymantriae* in the larval environment, did not influence the transmission of *N. lymantriae* (Goertz & Hoch, 2013). This was interpreted to be due to the high amount of spores already released from live *L. dispar* larvae by feces. We conclude that the transmission of *N. lymantriae* is likewise not affected by *F. fusca*. Our results do not indicate a negative effect of *F. fusca* on the transmission of *V. disparis* either. Workers of *F. fusca* avoided *Vairimorpha*-infected larvae when they had the choice between uninfected and infected larvae and both predation experiments did not result in a higher or lower proportion of infected test larvae compared to treatments without ants. Our results do not indicate a shortening of the pathogen's latent period, therefore no enhanced transmission of *V. disparis* occurs through predation by *F. fusca* unlike what was shown for *C. sycophanta* (Goertz & Hoch, 2013). This beetle contaminated the environment of *L. dispar* larvae when feeding on infected prey; spores were released from the host earlier than without predation. Our experiment with *F. fusca* does not indicate the removal of latently infected test larvae by ant workers, thus no evidence was obtained for removal of spore inoculum from the host's environment. We conclude that the transmission of *V. disparis* is not affected by *F. fusca*.

In summary, we showed that uninfected and infected *L. dispar* larvae are a potential prey for *F. fusca*. Horizontal transmission of both microsporidian species is not affected by the action of *F. fusca*. The predator never acquired microsporidiosis from infected prey.
